# An Autopsy Case Report: Prune Belly Syndrome with Overlapping Presentation of Partial Urorectal Septum Malformation Sequence

**DOI:** 10.5146/tjpath.2018.01440

**Published:** 2020-01-15

**Authors:** Çiğdem Kunt İşgüder, Mine Kanat Pektaş, Doğan Köseoğlu, Şahin Takçı

**Affiliations:** Department of Obstetrics and Gynecology, Istanbul University, School of Medicine Istanbul, Istanbul, Turkey; Department of Kocatepe University, School of Medicine, Afyon, Turkey; Department of Pathology, Gaziosmanpasa University, School of Medicine, Tokat, Turkey; Department of Pediatrics, Gaziosmanpasa University, School of Medicine, Tokat, Turkey

**Keywords:** Congenital anomaly, Newborn, Prune belly syndrome, Urorectal septum malformation sequence

## Abstract

Prune belly syndrome (PBS) is characterized by a classical triad of congenitally absent abdominal muscles, bilateral cryptorchidism, and a malformed urinary tract. Urorectal septum malformation sequence (URSM) is identified with the absence of urogenital and anal openings. This case report describes a 15-week-old female fetus with megacystis, ascites and oligohydramnios in a 19-year-old nulliparous woman. The patient underwent preterm labor at the 33rd gestational week and delivered a female newborn weighing 2250 grams who died three days later due to progressive respiratory insufficiency. To the best of our knowledge, this is the third case of an overlap between PBS and URSM in literature. Such an overlap refers to the existence of left renal agenesis, right renal cystic dysplasia, bilateral club foot and lumbar scoliosis as well as the absence of abdominal wall muscles, internal genital organs, urethral, vaginal and anal openings. This case report aims to remind the obstetricians about the concurrent occurrence of PBS with URSM and its poor prognosis.

## INTRODUCTION

Prune belly syndrome (PBS) is a congenital syndrome which is also known as Eagle-Barrett syndrome, Obrinsky syndrome, Fröhlich syndrome or abdominal muscular deficiency syndrome with a rare incidence of 1:26000 to 1:40000 births (1) .

Approximately 97% of the affected cases are male newborns who demonstrate a classical triad of congenitally absent abdominal muscles, bilateral cryptorchidism, and a malformed urinary tract. A deficiency in the development of abdominal muscles causes the typical wrinkling of the overlying abdominal skin and such an appearance is usually the first diagnostic clue. In fact, prune belly syndrome is a multisystem disease which leads to concurrent cardiopulmonary, gastrointestinal and musculoskeletal anomalies in varying morphological features (2).

This syndrome may be also observed in female newborns but naturally cryptorchidism does not exist in these newborns. Since affected female newborns do not have the classical findings of this syndrome, they are often termed as “pseudoprunes.” For instance, prune belly syndrome was identified in 5 female newborns and 13 male newborns in over half a million consecutive life births were detected in British Colombia from 1964 to 1978 (3).

The urorectal septum malformation sequence (URSM) emerges as a result of a defect in the caudal mesoderm. In other words, the urorectal septum fails to fuse with the cloacal membrane and ambiguous genitalia so that urogenital and anal openings do not appear and lumbosacral abnormalities occur. This clinical entity is also called female pseudohermaphroditism with caudal dysgenesis and cloacal dysgenesis sequence, and it is observed in one in 50000 to 250000 neonates. Although the complete form of this sequence is a lethal defect, its partial form has been reported to be compatible with life (4–6).

This case report describes a female newborn with an overlapping feature of PBS and URSM.

## CASE REPORT

A 19-year-old nulliparous woman presented to the study center for a routine pregnancy follow-up. It was learnt that she was at her 15th gestational week according to her last menstrual period. There was nothing particular in her medical history and there was no consanguinity between her and her spouse.

Obstetric ultrasonography showed a 15-week-old female fetus with enlarged urinary bladder which measured 30 mm in its longest diameter. Amniotic fluid index was normal and no additional sonographic finding was recorded. However, fetal megacystis, fetal ascites and oligohydramnios were visualized by obstetric ultrasonography two weeks later. The couple was informed about the possible poor prognosis of the fetus, but they decided to continue with the pregnancy. Thus, the patient was included in weekly follow-up program.

At the 33rd week of gestation, the patient was admitted to the study center due to preterm labor and premature rupture of membranes. On the same day, she delivered a female newborn weighing 2250 grams with a first minute Apgar score of 3 and a fifth minute Apgar score of 5. Initial physical examination revealed absent abdominal wall muscles, bilateral club foot, imperforate anus and ambiguous genitalia which could be described as a phallus-like perineal structure without urethral or vaginal openings. Due to the existence of intercostal and subcostal retractions, the newborn was transferred to the neonatal intensive care unit and application of high frequency oscillatory ventilation was started. Chest and abdominal X-ray showed an abnormal bell-shaped thoracic cage, bilateral pulmonary hypoplasia, decreased ventilation in both lungs, lumbar scoliosis and a distended abdomen. Abdominal ultrasonography demonstrated agenesis of the left and cystic dysplasia of the right kidney and absence of internal genital organs. Karyotyping revealed the presence of normal 46, XX chromosomes. Despite the drainage of urinary bladder through an umbilical catheter, the newborn was lost on the third day due to progressive respiratory insufficiency.

**Figure 1:**
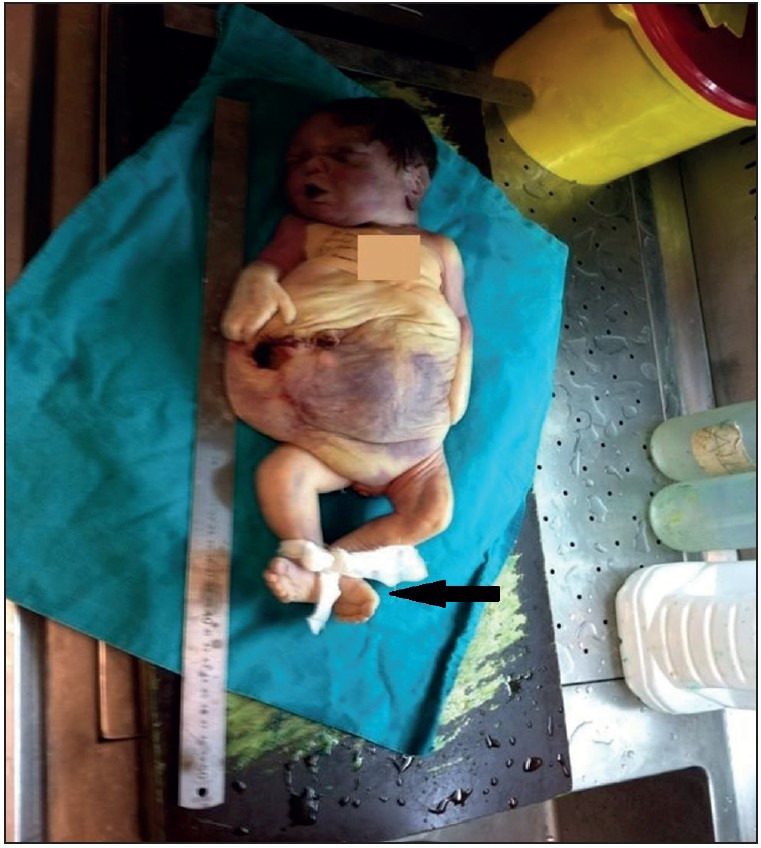
The newborn with bilateral club foot (arrow), and without abdominal wall muscles.

**Figure 2:**
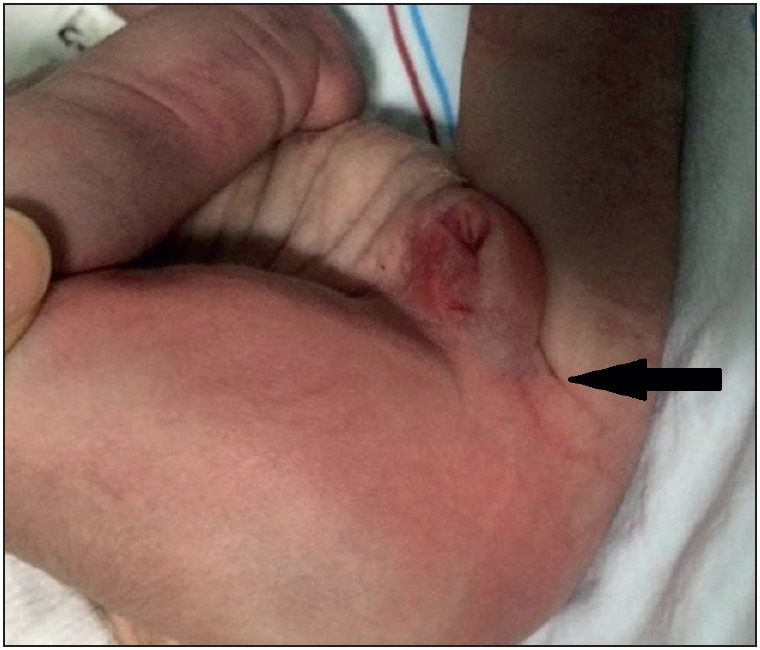
The affected newborn had imperforate anus (arrow) and a phallus-like perineal structure without any openings.

**Figure 3:**
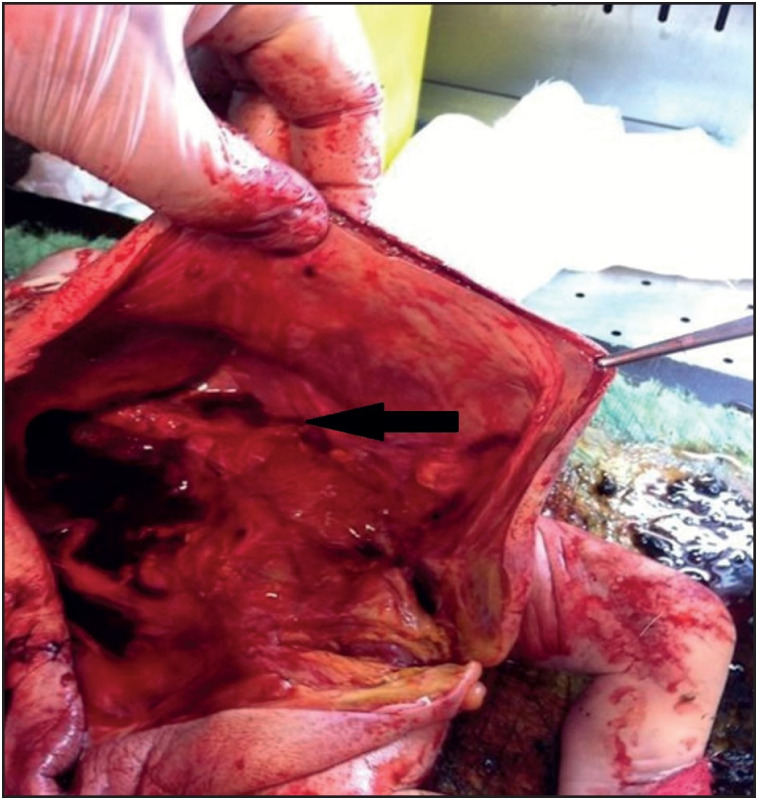
The affected newborn had left renal agenesis (arrow), flattened urinary bladder and absence of internal genital organs.

Permission for autopsy was obtained from the family. In addition to clinical and radiological findings, the foetus of 43 cm length showed potter’s facies with manifestations of ocular hypertelorism, low-set ears, flattened nose and receding of the chin. There was cystic dilatation of the abdomen with deficient development of abdominal muscles, and defective insertion of the umbilical cord in the anterior abdomen. In addition, club feet were observed (Figure 1). The anal orifice was absent (Figure 2). Intraabdominal exploration disclosed a bladder measuring 12x9 cm. The bladder was filled with meconium, and an anastomosis between the 58-cm-long bowels and the bladder was observed. Both internal and external layers of the bladder were flattened, and increased bladder wall thickness was noted. No tumor was encountered. The urethra was undetectable. At the right side, an ureteral orifice was detected. Internal genital organs were not detected in the pelvic and abdominal regions (Figure 3). At the right side, a cystic and dilated kidney in its normal anatomical position, and an adrenal gland with normal dimensions were observed. The liver, stomach, spleen, pancreas, and heart were in their normal anatomical location, and no cardiac anomaly was seen. The lungs had a normal number of lobes.

## DISCUSSION

Prune belly syndrome has been associated with cardiopulmonary, gastrointestinal and orthopedic abnormalities in 75% of the affected cases. For instance, club foot, pulmonary hypoplasia, potter’s facies, imperforate anus and arthrogryposis have been detected in 45%, 45%, 27%, 27%, and 18% of the patients with prune belly syndrome, respectively (1,3).

Moreover, urologic abnormalities such as urethral hypoplasia or atresia have been specified in around 18% of the cases and these abnormalities have been addressed as an independent risk factor for increased mortality. On the other hand, partial URMS has been associated with vertebral abnormalities, sacral agenesis/hypoplasia, single umbilical artery, limb anomalies, tracheoesophageal fistula and cardiac anomalies in 56%, 47%, 37%, 25%, 18% and 16% of the cases, respectively. Additionally, renal agenesis and dysplasia have been found in 50% and 82% of the patients with URMS, respectively (4–6).

The concurrent occurrence of PBS and URMS is an extremely rare clinical entity. Goswami et al. were the first to report a case of PBS with URMS (7). A nonviable female fetus which had a protruded abdomen and ambiguous genitalia was delivered at the 32nd week of pregnancy. On autopsy, the fetus was found to have female internal genital organs but her left kidney, urinary bladder and the rectum were absent. The sigmoid colon, the ureters and the fallopian tubes opened into a common cloacal sac and the histopathological examination of the ovaries indicated the presence of Leydig cells. Later, Farooqui et al. described a 34-week-old female twin fetus with absent anterior abdominal wall muscles, hydrometrocolpos, distended urinary bladder, moderate hydronephrosis, hydroureter, and linear streaked calcifications in the hypoplastic left kidney (8). Despite several surgical interventions, this newborn was lost at the third postpartum month.

To the best of our knowledge, this is the third case of an overlap between PBS and URSM in the literature. Such an overlap was characterized with the existence of unilateral renal agenesis and unilateral renal cystic dysplasia as well as the absence of internal genital organs and urethral, vaginal and anal openings. Bilateral club foot and lumbar scoliosis were the accompanying abnormalities.

The etiopathogenesis of prune belly syndrome or urorectal septum malformation sequence is still undetermined. It has been hypothesized that genetic mutations (i.e., deletion of hepatocyte nuclear factor-1-beta gene at 17q12) or familial predisposition (X-linked autosomal recessive mode of inheritance) may lead to PBS (1,6). The etiology of URSM has been linked to defective mesodermal proliferation during early embryonic development. It has been proposed that the severity of this sequence is related to the gestational age at which the developmental defect occurs. *In vivo* knockdown of Brachyury (a key regulator of mesoderm formation during early development) can cause anatomical malformations including skeletal defects, imperforate anus and ambiguous genitalia (4,6). As for the present case, the affected fetus had a normal karyotype of 46, XX but the aforementioned genetic mutations could not be investigated due to technical inadequacy. There was also no history of familial predisposition.

The treatment modalities of PBS include kidney transplantation, abdominoplasty and corrective surgery for undescended testes and malformed urinary tract. However, the prognosis of PBS is poor because of oligohydramnios-related pulmonary hypoplasia (3). The prognosis of partial URSM is also poor and several sessions of corrective surgery are scheduled if the newborn survives. In this case, the newborn was lost due to progressive respiratory insufficiency in her third day (6). The description of such a case aims to remind the obstetricians of the concurrent occurrence of PBS with URSM and its poor prognosis.
